# Mechanisms by which adverse childhood experiences, other traumas and PTSD influence the health and well-being of individuals with eating disorders throughout the life span

**DOI:** 10.1186/s40337-022-00696-6

**Published:** 2022-11-14

**Authors:** Timothy D. Brewerton

**Affiliations:** Department of Psychiatry and Behavioral Sciences, Medical University of South Carolina, Charleston, SC USA

**Keywords:** Trauma, Adverse childhood experiences, Eating disorders, Posttraumatic stress disorder, Comorbidity, Genetics, Environment, Epigenetics, Treatment, Outcome

## Abstract

**Background:**

Multiple published sources from around the world have confirmed an association between an array of adverse childhood experiences (ACEs) and other traumatic events with eating disorders (EDs) and related adverse outcomes, including higher morbidity and mortality.

**Methods:**

In keeping with this Special Issue’s goals, this narrative review focuses on the ACEs pyramid and its purported mechanisms through which child maltreatment and other forms of violence toward human beings influence the health and well-being of individuals who develop EDs throughout the life span. Relevant literature on posttraumatic stress disorder (PTSD) is highlighted when applicable.

**Results:**

At every level of the pyramid, it is shown that EDs interact with each of these proclaimed escalating mechanisms in a bidirectional manner that contributes to the predisposition, precipitation and perpetuation of EDs and related medical and psychiatric comorbidities, which then predispose to early death. The levels and their interactions that are discussed include the contribution of generational embodiment (genetics) and historical trauma (epigenetics), social conditions and local context, the ACEs and other traumas themselves, the resultant disrupted neurodevelopment, subsequent social, emotional and cognitive impairment, the adoption of health risk behaviors, and the development of disease, disability and social problems, all resulting in premature mortality by means of fatal complications and/or suicide.

**Conclusions:**

The implications of these cascading, evolving, and intertwined perspectives have important implications for the assessment and treatment of EDs using trauma-informed care and trauma-focused integrated treatment approaches. This overview offers multiple opportunities at every level for the palliation and prevention of EDs and other associated trauma-related conditions, including PTSD.

## Plain English summary

Adverse childhood experiences (ACEs) and other traumatic experiences commonly occur in the great majority of individuals with EDs. The mechanisms by which ACEs and EDs emerge and interact with each other to lead to early death is reviewed from the perspective of the ACEs pyramid and the escalating cascade of problems that it depicts. The levels of the ACEs pyramid as they apply to EDs and their overlap with trauma-related disorders, especially PTSD, are explored and discussed and include 1) genetics (heredity) and historical trauma (epigenetics); 2) social and local context as predisposing and protective factors; 3) the ACEs themselves as precipitating factors; 4) disrupted development of the brain; 5) social, emotional and cognitive impairments; 6) the adoption of risky or self-harming behaviors; 7) the increased disease, disability and social problems that ensue; and 8) subsequent early death by suicide or fatal medical complications. A better understanding of how these mechanisms are interwoven with each other may lead to improved and integrated interventions at all points, including prevention, screening and treatment.

## Introduction

The role of traumatic experiences in the development of eating disorders (EDs) has been historically controversial [1–3] and mirrors the nature-nurture dilemma that has hindered a more complete understanding of the etiology of EDs, as well as of psychiatric disorders in general [4–6]. At this juncture it has been well established that both genetic and environmental factors contribute to the development of EDs, but the delineation of the interactions between these two major forces, which now also involves epigenetic factors, is constantly evolving and notoriously complex [4, 7–12].

The subject of this narrative review focuses on the environmental piece as it relates to traumatic experiences, especially those that arise during childhood and contribute to the precipitation and/or perpetuation of EDs and related comorbidities, both psychiatric and medical [13]. A narrative style was chosen for this review, which is intended to be a general, accurate, and extensive guide to what is already known about these interrelationships but to put them in a new perspective that has not heretofore been fully acknowledged and which will hopefully establish a more integrated theoretical framework for future research and clinical application. Of particular focus will be EDs that are concurrent with posttraumatic stress disorder (PTSD), the only psychiatric disorder that requires a traumatic event as an integral part of its criteria [14]. This is despite the fact that other psychiatric disorders also have a history of traumatic experiences identified as an important risk factor [15–17]. Notably, it has been reported that cumulative ACEs and childhood traumas are significantly associated with total PTSD symptoms [18–20]. Another recent study examining the relationship between ACEs and several psychiatric disorders using representative samples from the United States and Ireland found significant links between ACEs and major depressive disorder, generalized anxiety disorder, PTSD, and complex PTSD, which demonstrated an exceptionally strong dose–response relationship [21].

Although PTSD is technically defined as a dichotomous disorder, like all psychiatric disorders, it exists on a spectrum. Therefore, it is useful to view its symptoms on a continuum of severity, which can be measured using validated structured interview and self-report assessment instruments, e.g., the Clinician Administered PTSD Scale for DSM-5 (CAPS-5) [22], the PTSD Symptom Checklist for DSM-5 (PCL-5) [23], and/or the International Trauma Questionnaire (ITQ) for ICD-11 [24, 25] for adults and older adolescents. Corresponding measures for children and younger adolescents are also available, i.e., the Clinician Administered PTSD Scale for DSM-5 Child-Adolescent Version (CAPS-CA-5) [26] and the Child and Adolescent Trauma Screen 2 (CATS-2) [27]. Partial or subthreshold PTSD has been found to be of immense clinical importance to EDs and related psychiatric comorbidity [28–31]. This makes it imperative to assess for lifetime PTSD, not just the current form of the disorder, as lifetime PTSD and its symptoms have important predictive value for comorbidity and course of illness [15, 28, 32–34].

Other investigators have also shown that lifetime traumatic stressors and ACEs uniquely predict concurrent PTSD, complex PTSD, and the dissociative subtype of PTSD symptoms, whereas recent adult non-traumatic stressors do not [35]. Complex PTSD as defined by ICD-11 comprises all of the characteristics of classical PTSD, including intrusive, avoidant, and hyperarousal symptoms, in addition to disturbances in self-organization symptoms that are often characterized as personality disorders [24, 36–39]. Complex PTSD is typically associated with sustained, repeated, and multiple forms of traumatic experiences, which are typical in individuals with EDs, especially those in higher levels of care [13, 20, 40, 41]. It is important to note that PTSD and complex PTSD are major mediators and/or moderators of psychiatric comorbidity over and above the effects of traumatic events or experiences alone [42–45]. To fully appreciate the impact of trauma, the Substance Abuse and Mental Health Administration of the United States has emphasized the importance of the “three *E*’s,” i.e., *E*vent(s), *E*xperience(s), and *E*ffect(s) [46, 47]. Thus, the focus of this narrative review is on elucidating the mechanisms involved in mediating the *E*ffects of an individuals’ *E*xperiences of traumatic *E*vents. Specifically, the *E*ffects of interest include PTSD, other trauma-related comorbid disorders, EDs and their interrelationships. Such trauma-related comorbid disorders include but are not limited to mood, anxiety, dissociative, substance use, disruptive/impulse control, obsessive–compulsive, attention-deficit hyperactivity disorder (ADHD) and personality disorders, all of which are also commonly seen in individuals with EDs [14, 48–53].

Recent network analyses show significant links between PTSD and EDs, which has further strengthened the realization of their robust interconnectedness and underscores the need to more carefully delineate their underlying, interacting mechanisms [54–56]. As noted by Liebman and colleagues, results from network analyses “provide preliminary support for conceptualizing PTSD-ED comorbidity as a truly comorbid trauma-related syndrome rather than two distinct co-occurring disorders” [55]. How the underlying etiological processes of this entity unfold over the course of human development to create this “system of causally related and reciprocally reinforcing set of symptoms” [54] needs further clarification and will be useful to clinicians and researchers alike. It is from this perspective that this review will attempt to demonstrate the interwoven nature between ACEs, other traumas, their origins and sequelae, and EDs.

## Methods

**Fig. 1:**
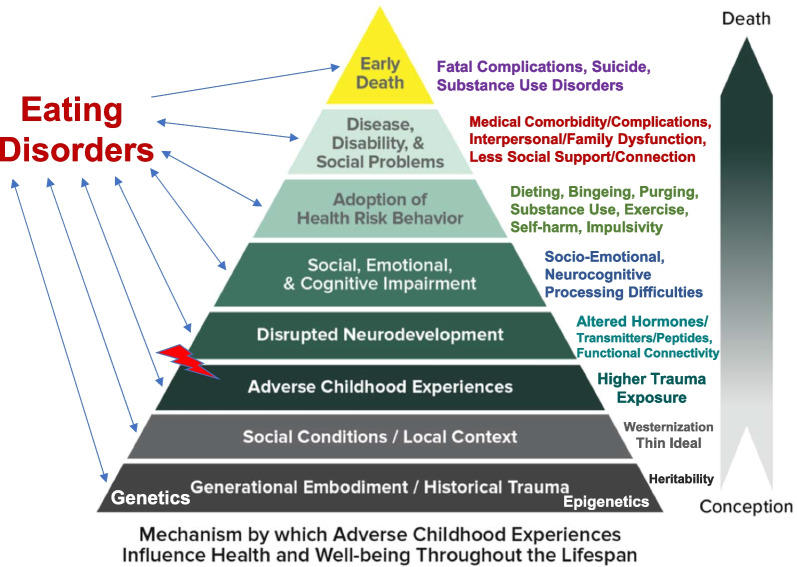
Mechanisms by which adverse childhood experiences (ACEs) and other traumas influence the health and well-being of individuals with eating disorders throughout the life span. The ACE pyramid (Centers for Disease Control and Prevention, cdc.gov) is reproduced with permission and adapted to eating and related disorders. Available via license: https://creativecommons.org/licenses/by/3.0/legalcode

The structure of this literature review will be based on the well-known ACEs pyramid created by the Center for Disease Control and will progress from a discussion of the bottom level of the pyramid, i.e., generational embodiment and historical trauma, to the top level, i.e., early death (see Fig. 1). This analysis will take the form of a narrative review, which seeks to examine and integrate a diverse collection of quantitative studies that have used disparate methodologies and diverse theoretical orientations, conceptualizations, and/or constructs [57, 58]. This methodology is particularly useful as a means of linking together studies on different topics for reinterpretation or interconnectedness in order to develop a more comprehensive, intertwining, and overarching synthesis [59]. A systematic review was not deemed to be suitable for this paper, since there is no one well-defined focus of review but rather multiple, interwoven levels of understanding.

Evidence from the scientific literature will be cited that supports the contention that EDs interact with each of these proclaimed biopsychosocial and psychoneurobiological mechanisms in an interactional, reciprocal manner that contributes to the predisposition, precipitation and perpetuation of EDs and related medical and psychiatric comorbidities, which then result in heightened morbidity and mortality.

### Components of the ACEs pyramid

I. **Generational embodiment (genetics) / historical trauma (epigenetics)**
  Twin studies substantiate the powerful hereditary forces predisposing toward the development of EDs, including anorexia nervosa (AN), bulimia nervosa (BN), binge eating disorder (BED), and purging disorder (PD) [7, 11, 60–63]. Reliable estimates of the variance accounted for by additive genetic factors are on the order of 40–60% for AN, BN and BED. In addition, genetic factors also highly contribute to the development of PTSD and dissociation [64–69]. In a study of 400 same-sex twin pairs examining the genetic overlap between PTSD symptoms and maladaptive eating behaviors, heritability estimates were 48% for PTSD symptoms and 45% for eating behaviors. Notably, the investigators found genetic correlations of 34% between PTSD symptoms and eating behavior overall and of 53% between PTSD symptoms and uncontrolled eating [70], thereby suggesting common genetic predispositions.
  Recent studies reveal that the propensity to experience and to report child maltreatment may have some genetic basis in part [71–73]. Furthermore, agreements between retrospective and prospective reports of child maltreatment are significantly correlated but to a low degree [74].
  Matters are further complicated when one considers that the effects of historical or generational traumas, including the impact of widespread famine occurring in previous generations, can be highly influential on subsequent progeny via epigenetic mechanisms [75–84]. Such effects are reported to occur even when the individual has not had direct contact with the trauma itself or the traumatized relative [85–89]. DNA methylation, which is a measure of epigenetic change and which ostensibly mediates such effects, has been associated with a number of changes in mental illness phenotypic manifestations, including brain structure and function, that are relevant to EDs, PTSD and other trauma-related disorders [76–78, 90–99]. Significantly, DNA methylation alterations in AN have been shown to be indicative of chronicity and illness severity but can be ‘reset’ and normalize with treatment and remission of illness [100].
II. **Social conditions / local context**
  The social environment and local or cultural context are known to influence exposure to child adversity and the development of PTSD and other adverse consequences [101–106]. These may include poverty, social disorganization and social disadvantage at the community or neighborhood level, racial disparities, exposure to political and/or family violence, various types of stigmatizations, lack of educational, medical and other support services, and generally living in unsafe environments. Likewise, social environment and local or cultural context are important contributors to the development of EDs [107–115]. Some of the most noted risk factors include Westernization, industrialization, urbanization, modernization, migration, thin idealization and associated body dissatisfaction and sexual objectification of women [111, 113, 116–120]. It is well established that EDs occur in all ethnicities and socioeconomic classes, and all ethnicities and socioeconomic classes are at risk for child maltreatment and traumatic experiences [102, 121]. Therefore, it is reasonable to assume that there are overlapping sociocultural and environmental risk factors that contribute to trauma, trauma-related disorders, and EDs [122–133].
  Sociocultural factors are thought to play important roles in the development of EDs of all types [121]. However, twin studies generally indicate that shared environmental factors do not substantially contribute to the development of AN and BN, although they may play more of a role in BED and PD. Overall, it appears that nonshared environmental factors account for a much greater share of the variance that explains ED development than shared environmental factors [7]. Likewise, twin studies of PTSD also show a substantial contribution of nonshared environmental factors, which includes exposure to traumatic events [66, 134].
  There is a rich literature demonstrating important family environmental factors that appear to contribute to victimization and the development and/or maintenance of EDs and PTSD [135–141]. In addition, alterations in parental sensitivity and impaired attachment in parents with PTSD to their children are other important considerations when evaluating the role of social and familial contexts in mediating the effects of adversity on the health and well-being of individuals with EDs [78, 79, 142–144]. Parents of hospitalized ED patients have been found to have high rates of posttraumatic stress symptoms, which were associated with parental mood symptoms, avoidance, symptom accommodation, and inflexibility [145]. Furthermore, high degrees of expressed emotion (EE index) have been reported in the family members of patients with EDs [146, 147] and PTSD [148, 149] and are associated with relatively worse outcomes. Conversely, social support and other pro socio-interpersonal factors can be protective and foster resilience and recovery [150–160].
  Another important set of environmental factors that may influence the occurrence of ACEs and other traumatic events include meteorological variables, such as season, weather and climate, which are known to influence the expression of aggressive behaviors, including violence toward self and others (both adults and children) [161–170], as well as the clinical expression of EDs [171–179], PTSD and other trauma-related disorders [180–182]. Much evidence has identified seasonal changes in serotonergic and dopaminergic function as mediators of these phenomena via the effects of seasonal changes in the photoperiod, temperature, and relative humidity on human biological systems [183–186].
III. **Adverse childhood experiences**
  Several studies attest to the association between the cumulative number and severity of ACEs endorsed and resultant diagnoses of PTSD, complex PTSD, and other trauma-related psychiatric comorbidities, including mood, anxiety, dissociative, personality, and substance use disorders (SUDS) [18, 19, 21, 35, 187–192]. Evidence for the association between EDs and prior traumatic experiences, especially during childhood, is now also incontrovertible. In comprehensive meta-analyses of the role of childhood maltreatment in EDs, all types of childhood maltreatment have been associated with all types of EDs, especially those with features of binge eating and/or purging [13, 193].
  Longitudinal studies and cross-sectional studies using national representative samples, case control studies, and samples of individuals receiving treatment show that stressors and traumas of various types are associated with EDs [20, 40, 48, 194–212]. These include sexual abuse, physical abuse, emotional abuse, physical neglect, emotional neglect, bullying, harsh physical punishment, parental divorce and mental illness, any type of family dysfunction, as well as exposures to intimate partner violence, transportation accidents, toxic substances, captivity, life threatening illness or injury, severe human suffering, sudden violent or accidental death, or any other very stressful event. In addition, *causing* serious injury, harm or death can be also traumatic for ED patients and lead to PTSD [40]. A dose–effect relationship has been reported to characterize the number of childhood trauma types and the severity of ED clinical features, as well as worse quality of life and mental well-being [13, 40, 213–215]. These data suggest a consistent and independent association between childhood maltreatment and more severe clinical and functional characteristics in EDs.
  In another study, children and adolescents with credible, substantiated histories of childhood sexual and/or physical abuse following forensic assessment showed higher ED symptomatology than those with non-credible, non-substantiated disclosures, and linear regression indicated that this finding was mediated by PTSD and dissociative symptomatology [216]. Similarly, other investigators have found that child maltreatment, and especially the presence of PTSD or PTSD symptoms, are powerful predictors and mediators of ED development and chronicity [30, 122, 123, 214, 217–219].
  Additionally, using a national representative sample, the odds of having an ED diagnosis of AN or BED were found to be significantly higher in respondents with a history of childhood food neglect compared with those without such history [220]. Other studies have also linked emotional neglect, physical neglect, and food insecurity with EDs and their severity [204, 205, 221–224]. Notably, food insecurity is also significantly associated with other forms of traumatic events and greater degrees of PTSD symptoms, depression, generalized anxiety, and substance use [225–228].
  Just as traumatic experiences may predispose to the precipitation and perpetuation of eating and related disorders, eating and related disorders may also predispose to experiencing more traumas [229–232]. It is well-established that once an individual has been sexually assaulted the first time, the chances of experiencing a subsequent sexual assault significantly increase [233]. This phenomenon may in part be mediated by higher levels of impulsivity, disinhibition, dissociation, substance use, and/or other self-destructive tendencies, which are more likely associated with binge-type EDs as well as PTSD itself [127, 231, 232, 234–239]. In addition, the lack of an accurate appraisal or appreciation of dangerous situations as well as the propensity to repeat or reenact traumas has been postulated to contribute to traumatic recurrence [240–242].
  It is also important to note that prenatal and perinatal factors, such as prematurity and obstetric complications resulting in hypoxia, may predispose to the development of EDs and other psychiatric sequelae, even though these are not customarily thought of as ACEs [243–248]. In addition, maternal history of child maltreatment and depression during pregnancy is associated with subsequent child maltreatment, depression, and other internalizing and externalizing difficulties in the children of such mothers [75, 249]. Likewise, stress exposure during the third trimester of pregnancy predicts later ED symptoms in children at age thirteen [250]. Such early insults may “prime the pump,” so to speak, and sensitize the individual to become more vulnerable to subsequent traumas later in life. Altered or enhanced stress sensitivity can be genetically programmed as well as environmentally induced through sensitization of the nervous system and potential epigenetic changes, which have been discussed above [251].
IV. **Disrupted neurodevelopment**
  Adverse childhood experiences have been found to produce profound and lasting changes in the structure and function of the human central nervous system (CNS) [252–254]. EDs, PTSD, and other trauma-associated psychiatric sequelae, are characterized by complex disruptions in neurodevelopment over the course of a lifetime. Alterations in the autonomic nervous system and the hypothalamic–pituitary–adrenal (HPA) axis associated with traumatic stress initiate a complex cascade of downstream neurobiological effects that are shaped by the environment and that have been well described in the scientific literature [76, 77, 255–261]. Likewise, the role of stress in the pathophysiology of EDs has also been underscored and involves similar changes [262, 263]. Furthermore, alterations in stress reactivity have been reported in trauma-related disorders, including EDs, particularly those with dissociation [255, 264–267]. Individuals with both EDs and a history of childhood maltreatment have been reported to have unique neuroendocrine signatures characterized by delayed morning cortisol awakening, a finding that has been also reported in association with child maltreatment and PTSD, especially when chronic [268–273]. Such neuroendocrine deviations have been linked to altered stress responsivity as well as to altered emotional regulation, leading to the postulation of a “maltreated ecophenotype” to describe these individuals [268, 269, 274]. This designation has also been applied to non-ED individuals with histories of child maltreatment and/or PTSD, who are purported to demonstrate distinct clinical and neurobiological features [270].
  Alterations in a variety of hormones, neurotransmitters, and neuropeptides have been well described in the literature for both EDs and PTSD, and a comprehensive overview of these findings is beyond the scope of this paper [275–278]. However, these alterations involve a number of disturbances in the interplay of several different stress-responsive systems that interact centrally and peripherally to produce individualized clinical phenotypes and vulnerabilities.
  Alterations in functional connectivity have been investigated in separate studies of both PTSD [279–282] and ED patients [283–286]. No such studies have yet been reported in ED patients with and without PTSD. However, one study has reported on the effects of childhood maltreatment on brain structure in adults with EDs and has shown reduced grey matter volume in the right paracentral lobule and in the left inferior temporal gyrus [287]. There was also reduced white matter integrity in the corpus collosum, internal capsule, posterior thalamic radiation, longitudinal fasciculus and corona radiata of those with child maltreatment. Negative correlations were also found between white and grey matter changes and Childhood Trauma Questionnaire emotional and physical neglect scores.
  Developmental disruptions in the neurobiology of individuals with childhood trauma or PTSD underlie reported clinical psychopathology and may predict treatment outcome [288–293]. It has been noted that ACEs and other traumas in the context of EDs results in greater severity of illness, more binge-purge features, higher psychiatric comorbidity, more suicidality, and increased refractoriness to treatment [13, 294]. Likewise, the presence of PTSD in patients with EDs is also associated with greater severity of illness, greater psychiatric comorbidity, greater prevalence of somatic symptoms, worse quality of life, and worse outcomes [20, 32, 40, 214, 295–297]. Essentially, trauma has been described as being “biological embedded,” a term which very much applies to individuals with both EDs and PTSD [298–302]. In summary, such biological embedding involves chronic disruption in a variety of interrelated neurodevelopmental pathways, including activation of the HPA axis, increased allostatic load, enhanced inflammation, altered reward sensitivity, consequential epigenetic changes, as well as profound structural and functional changes in the brain [303–306]. These pathways are in turn theorized to lead to EDs, food addiction, and SUDs, each of which have been linked to obesity and other related comorbidities [298, 299, 307–312].
V. **Social, emotional and cognitive impairment**
  Traumatic sequelae as a result of maltreatment, including PTSD, involve impairments in the ability to process social, emotional and cognitive information [313–320]. Such deficits may include the identification and verbalization of one’s own emotional state of mind, i.e., alexithymia, as well as that of others. Several types of socioemotional, neurocognitive, and processing difficulties have been demonstrated in EDs at all phases of the illness and may (1) impede the processing of traumatic events; (2) lead to the misidentification of potentially threatening interpersonal situations; and (3) interfere with receiving quality emotional support from others that is so crucial to healing and recovery [154, 315, 321–342]. Such deficits and related interpersonal dysfunction may play significant roles in predicting treatment outcomes and responses to treatment for both EDs and PTSD, as well as their combination [150, 326, 343–349]. Future studies that examine social, emotional and cognitive functioning in ED patients with and without prior ACEs, as well as those with and without PTSD symptoms, would be illuminating.
  Socioemotional processing difficulties have been identified as a central focus of treatment for EDs as exemplified in the Maudsley Model of Anorexia Nervosa Treatment for Adults (MANTRA) [350, 351]. Likewise, treatments for PTSD, such as cognitive processing therapy (CPT), often attempt to directly target social role or interpersonal processes [352]. Taken together, there is much evidence for a “social ecology” for both PTSD and EDs within which lies a conceptual framework for understanding how risk and recovery is highly dependent on social phenomena or the processing of social phenomena [150, 152, 156].
VI. **Adoption of health risk behavior**
  It is generally accepted that ACEs, in a dose-dependent fashion, lead to self-destructive behaviors, including substance use and the adoption of other health risk behaviors, such as poor self-care, outright self-harm and suicidality, as well as ED behaviors [13, 353, 354]. All of the ED behaviors, including food restriction, binge eating, purging, compulsive exercising, and the abuse of substances to manage weight and emotional dysregulation, are potentially harmful and life-threatening behaviors [311, 355, 356]. All ED behaviors alter neurochemistry, including monoaminergic neurotransmitter systems and associated reward mechanisms that mediate related clinical symptomatology and reinforce repetition of risky behaviors [127, 278, 357–364].
  Binge eating, purging and substance abuse are ED-related behaviors that are particularly associated with stressful life events, childhood maltreatment and PTSD [13, 251, 365–370]. Suicidal behavior and other forms of impulsive self-injurious behaviors are also common in eating disordered individuals with histories of abuse and trauma-related disorders [371–375].
  Taken together, the adoption of health risk behaviors by traumatized ED patients is a natural outcome of ACEs, their biopsychosocial contributions (genetics, epigenetics, social/local contexts), their resulting disruption of neurodevelopment and consequent impairment in social, emotional and cognitive processing.
VII. **Disease, disability, and social problems**
  As a direct result of the adoption of health risk behaviors that become habitual, repetitive, and then chronic, there is an eventual and inevitable increase in the rates of disease, related disabilities, and poorer quality of life. A host of medical and psychiatric comorbidities, including eating, substance use, mood, anxiety, dissociative, and personality disorders, have been reported to be significant outcomes of ACEs, other traumatic experiences, and PTSD [15, 18, 20, 40, 128, 190, 212, 299, 376–384]. These trauma-related conditions and disorders are widespread and systemic and involve multiple bodily systems, including the cardiovascular, endocrine, gastrointestinal, musculoskeletal, autoimmune, and autonomic and central nervous systems [298, 353, 376, 377]. Eating disorders are also fraught with medical complications and comorbidities in multiple systems that are known to impair quality of life even when they don’t involve traumatic histories [355, 385–387]. However, impaired quality of life in multiple domains, including psychological, physical-cognitive, financial, and work-school, are significantly more severe in eating disordered individuals with PTSD compared to those without PTSD [20, 40]. Likewise, there is significantly greater severity of ED, major depression and anxiety symptoms in those with PTSD compared to those without [20, 40, 388].
  Substance use disorders (SUDs) are known to be related to maltreatment/trauma, PTSD, and EDs, and the contribution of SUDs to the development of disease, disability and social problems can be substantial [15, 251, 370, 389–393]. Notably, the presence of both SUD and ED has been associated with higher rates of child maltreatment, greater severity of PTSD, depression, and other psychopathological features, less responsiveness to treatment, and greater mortality [394–400].
VIII. **Early death**
  ACEs are not only associated with a distinctly lower health-related quality of life and an enhanced burden of disease, but they also predict lower life expectancy [401]. EDs are also associated with enormously high social and economic cost due to their high burden of disease [402–404]. Higher rates of premature death are parts of the landscape for EDs, ACEs, and PTSD, as well as other trauma-related disorders, including substance-related and addictive disorders [405–409]. ACEs and PTSD result in early death from a variety of causes that are mediated by various stress pathways but which may ultimately result in telomere shortening, which in turn is linked to premature aging and earlier mortality [377, 380, 410–414]. Likewise, EDs are also known to have high mortality rates, which results from the direct sequelae of starvation and other ED behaviors or suicide that is often associated with SUDs, especially alcohol use disorder, and other psychiatric comorbidity [386, 415–426]. To the extent that higher doses of trauma and PTSD contribute to increased mortality, this is directly relevant to individuals with EDs, particularly those with concurrent PTSD, earlier onset of illness, higher rates of psychiatric comorbidity, and relative treatment refractoriness [214, 219].

## Conclusions

Using the ACEs pyramid as a basis, the mechanisms by which ACEs and other traumatic experiences arise and may lead to a variety of comorbid conditions and early death is reviewed for both EDs and posttraumatic symptomatology, especially PTSD and other related conditions. The ACEs pyramid provides a helpful scaffolding with which to understand the mechanisms that ACEs and other traumas occur and influence the health and well-being (or lack thereof) of individuals with EDs throughout the life span. These levels of mechanisms include (1) generational embodiment and historical trauma, (2) social and local context as predisposing factors, (3) the ACEs themselves as precipitating factors, (4) disrupted neurodevelopment; (5) social, emotional and cognitive impairment; (6) the adoption of health risk behaviors; (7) increased disease, disability and social problems, and (7) subsequent early death. A better understanding of these mechanisms in more depth that mediate the interaction between ACEs, other traumas, and EDs presents enhanced opportunities for possible intervention.

The treatment implications of these concepts are numerous and include primary, secondary and tertiary prevention efforts at all levels of the pyramid. The importance of the prevention of child maltreatment must be embraced by all of psychiatry and mental health, including ED professionals. It is also highly recommended that ED clinicians and/or treatment centers thoroughly screen for ACEs and other traumatic experiences and their effects in all patients. Trauma is a universal problem and trauma-informed care has become the standard of care in all behavioral health care services, including those for eating and related disorders [46, 128, 384]. Despite any potential misgivings or risks, ED professionals would do well to acquaint themselves with evidence-based, trauma-focused treatment approaches, such as integrated cognitive behavioral therapy for EDs and PTSD [427–429].

A potential major limitation of this review is that it is narrative in style and largely represents the views of a single author. However, a systematic review was not deemed suitable for this project, since there is no one well-defined focus of review. Rather, this paper attempts to present a broad overview regarding the multiple levels of the ACEs pyramid and their potential multidirectional interrelationships to EDs, PTSD, and other trauma-related disorders. Every effort has been made to cite the best supporting studies, many of which are systematic reviews and meta-analyses.

## Data Availability

Not applicable.

## Abbreviations

ACEs: Adverse childhood experiences
AN: Anorexia nervosa
BN: Bulimia nervosa
BED: Binge eating disorder
CPT: Cognitive processing therapy
EDs: Eating disorders
MANTRA: Maudsley model of anorexia nervosa treatment for adults
PTSD: Posttraumatic stress disorder
PD: Purging disorder
SUD: Substance use disorder
